# Association of the 36-Item Short Form Health Survey Physical Component Summary Score With Patient Satisfaction and Improvement 2 Years After Total Knee Arthroplasty

**DOI:** 10.1001/jamanetworkopen.2019.0062

**Published:** 2019-02-22

**Authors:** Bryon Jun Xiong Teo, Joyce Suang Bee Koh, Lei Jiang, John Carson Allen, Seng Jin Yeo, Tet Sen Howe

**Affiliations:** 1 Department of Orthopaedic Surgery, Singapore General Hospital, Singapore; 2 Centre for Quantitative Medicine, Duke-NUS Medical School, Singapore

## Abstract

**Importance:**

Increases in total knee arthroplasty (TKA) utilization rates suggest that its indications have been expanded to include patients with less severe symptoms. A recent study challenged the cost-effectiveness of TKA in this group of patients.

**Objective:**

To determine the association of the 36-Item Short Form Health Survey physical component summary score (SF-36 PCS) with patient satisfaction 2 years after TKA.

**Design, Setting, and Participants:**

This cohort study reviewed registry data from 2 years of follow-up of patients who underwent unilateral TKA from January 1, 2010, to December 31, 2014, at a single-center tertiary institution in Singapore. Data were acquired on April 27, 2017, and analyzed from August 15, 2017, to December 22, 2017.

**Main Outcomes and Measures:**

Patient satisfaction and SF-36 PCS. Preoperative disability and postoperative function as measured by the SF-36 PCS were correlated with Δ (2-year end point score minus baseline score) and patient satisfaction, scored on a 6-point Likert scale, with lower scores indicating greater satisfaction.

**Results:**

Of the 6659 patients, 5234 (78.6%) were female and 5753 (86.4%) were of Chinese ethnicity. Mean (SD) age was 67.0 (7.7) years, and mean (SD) body mass index (calculated as weight in kilograms divided by height in meters squared) was 27.7 (4.6). At 2-year follow-up, the mean (SD) SF-36 PCS score improved from 32.2 (10.1) to 48.2 (9.5) (*P* < .001). There were 1680 patients (25.2%) who described their satisfaction as excellent, 2574 (38.7%) very good, 1879 (28.2%) good, 382 (5.7%) fair, 96 (1.4%) poor, and 48 (0.7%) terrible. The minimal clinically important difference (change in SF-36 PCS of 10 from baseline) was met in 4515 patients (67.8%), and overall satisfaction was 97.8% (6515 patients). Covariance analysis showed significantly higher satisfaction in patients with preoperative scores of 40 to less than 50. Patients who were unlikely to meet the minimal clinically important difference compared with those who were likely to meet it (SF-36 PCS of ≥42.1 vs <42.1) had significantly higher 2-year satisfaction (mean [SD], 2.15 [0.9] vs 2.23 [1.0]; *P* = .009).

**Conclusions and Relevance:**

The findings suggest that a general health score, such as SF-36, is not associated with patient satisfaction 2 years after TKA. Functional assessment, preoperative counseling, and modification of expectations appear to remain vital before TKA.

## Key Points

**Question:**

Is the 36-Item Short Form Health Survey physical component summary score associated with patient satisfaction after total knee arthroplasty?

**Findings:**

In this cohort study of data from 6659 patients in Singapore, 67.8% of patients had minimal clinically important improvement in 36-Item Short Form Health Survey physical component summary score, but overall patient satisfaction was high at 97.8%.

**Meaning:**

The findings suggest that a general health score, such as the 36-Item Short Form Health Survey, may not be associated with patient satisfaction after total knee arthroplasty.

## Teaser

This cohort study of patients in Singapore undergoing total knee arthroplasty compares preoperative disability and postoperative function, as measured by the 36-Item Short Form Health Survey, with patient satisfaction 2 years after total knee arthroplasty.

## Introduction

Osteoarthritis is reported to be the 11th greatest contributor to disability globally and the 38th greatest in disability-adjusted life-years. This demand on health services is set to increase with the aging and increasing obesity of the world’s population.^1^ For patients with refractory knee osteoarthritis, total knee arthroplasty (TKA) remains an effective treatment with predictable outcomes compared with other treatment modalities.^2^ Losina et al^3^ reported that the increase in TKA utilization rates in the United States was not fully explained by population growth and obesity and suggested that the rapid increase among younger patients may be a result of expanding indications for this procedure.

In a recent publication, Ferket et al^4^ challenged the cost-effectiveness of TKA in this group of younger patients with less severe symptoms. By simulating a model, they reported that providing total knee replacement to patients with 12-Item Short Form Health Survey (SF-12) physical component summary (PCS) scores less than 35 was the optimal scenario, given a cost-effectiveness threshold of $200 000 per quality-adjusted life-years (QALYs) with a cost savings of $6974 and a minimal loss of 0.008 QALYs compared with the current practice. Restricting TKA to more severely affected patients (ie, those with lower preoperative scores) enabled the procedure to be more cost-effective than its current use.

Cost-effectiveness based solely on improvement in outcome scores may not adequately take into account the expectations and needs of the individual patient. Our study aimed to evaluate the association of the 36-Item Short Form Health Survey (SF-36) PCS with patient satisfaction after TKA by examining the association of preoperative disability and postoperative function as measured by the SF-36 PCS with its Δ (2-year end point score minus baseline score) and patient satisfaction scores.

## Methods

Prospectively collected institutional registry data were reviewed for all patients who underwent primary unilateral TKA for Kellgren-Lawrence grade 3 to 4 knee degenerate osteoarthritis at a single tertiary institution, Singapore General Hospital, from January 1, 2010, to December 31, 2014. On April 27, 2017, data from hospital electronic medical records were retrieved. Data analysis was conducted from August 15 to December 22, 2017. Patient demographics, side of surgery, and individual body mass index (BMI) (calculated as weight in kilograms divided by height in meters squared) were established from records retrieved. Race/ethnicity was assessed because it has been shown to have an effect on both preoperative scores and functional outcomes after primary TKA even after adjusting for confounders. We classified race/ethnicity according to self-reports by the patients.^5,6,7,8^ Preoperative scoring was determined at the preoperative evaluation to provide an accurate benchmark for comparison. We excluded patients with secondary arthritis from posttraumatic, inflammatory, or infective causes and those with postoperative prosthetic joint infections or fractures. Patients with these conditions are known to have a different prognosis from those with primary osteoarthritis. Different considerations (presence of long-term systemic medications) and surgical techniques are also needed to account for anatomical and physiological differences.^9,10^ This study was conducted with approval from the Singhealth Centralized Institutional Review Board, which waived informed consent because the study was a retrospective review of a large sample with no effect on or interaction with patient treatment. This study followed the Strengthening the Reporting of Observational Studies in Epidemiology (STROBE) reporting guideline.

The SF-36 scores for each patient were assessed preoperatively and at the 2-year follow-up. The 2-year mark was chosen as the end point because it approximates the time that early rehabilitation potential plateaus with concurrent reduction in the likelihood of the occurrence of an unrelated musculoskeletal event that could affect SF-36 scores at a longer follow-up. The SF-36 is a generic 36-item, patient-reported survey of health commonly used to determine care outcomes in adult patients.^11^ The survey contains 8 domains: vitality; physical functioning; bodily pain; general health perceptions; physical functioning; emotional functioning; social functioning; and mental health. Possible scores range from 0 to 100, with higher scores representing better health status. The SF-36 PCS was compiled from individual scores of the 8 domains using a formula validated for the study population. The SF-36 PCS was chosen as the primary variable because it has the advantage of lower inherent variability than the individual domain scores and allows elimination of both floor and ceiling effects.^12^ The difference in the SF-36 PCS (ΔPCS) after TKA was calculated by comparing SF-36 PCS score at 2 years after surgery with its preoperative value. Patients with a ΔPCS meeting or exceeding the minimal clinically important difference (MCID) of 10 were considered to have a clinically significant improvement after surgery.^13^ This cutoff was used to dichotomize patients into 2 groups: improvement and no apparent improvement. Based on the dichotomized MCID outcome as a response, a preoperative PCS cutoff was determined using receiver operating characteristic analysis as a diagnostic tool for predicting improvement or no improvement after surgery. Subgroup analysis was performed between patients with scores below and above the preoperative PCS cutoff to determine differences in patient characteristics and outcomes.

Patient satisfaction scored at the 2-year follow-up was recorded by an interviewer masked to previous scores. Satisfaction was scored on a 6-level Likert scale (1, excellent; 2, very good; 3, good; 4, fair; 5, poor; and 6, terrible). Adapted from question 53 of the North American Spine Society Questionnaire, the satisfaction question was specifically worded as, “How would you rate the overall results of your treatment for leg pain?” To reduce interviewer bias, this question was asked exactly as phrased in a neutral tone without further substantiation. A score of 4 or less was considered to be indicative of patient satisfaction.

### Statistical Analysis

Two-year patient satisfaction least-squares means were compared among preoperative SF-36 PCS groups using analysis of covariance with adjustment for age, BMI, sex, race/ethnicity, and side of surgery. A significant omnibus *F* test was followed by post hoc pairwise comparisons. We calculated 95% CIs for the mean differences. Univariate logistic regression was used to assess the association between preoperative SF-36 PCS score and dichotomized MCID. Receiver operating curve analysis was performed to identify a statistically optimal diagnostic cutoff based on the Youden *J* statistic. For this analysis, logistic regression was used. Spearman correlation was calculated for SF-36 vs ΔSF-36. Statistical analyses were performed using SAS software, version 9.4 (SAS Institute Inc). The level of statistical significance was taken to be 5% (2-sided test).

## Results

During the study period from January 1, 2010, to December 31, 2014, 6659 patients met the inclusion criteria for analysis (follow-up rate, 84.3%; 1243 patients were lost to follow-up). Among the 6659 patients who met inclusion criteria, mean (SD) age was 67.0 (7.7) years; 5234 (78.6%) were female; 5753 (86.4%) were of Chinese ethnicity; and mean (SD) BMI was 27.7 (4.6). Right-sided TKA was performed in 3456 patients (51.9%). Preoperative SF-36 PCS scores were banded into 5 groups (<20, 20 to <30, 30 to <40, 40 to <50, and ≥50). At 2-year follow-up, the mean (SD) SF-36 PCS score improved from 32.2 (10.1) to 48.2 (9.5) (*P* < .001). There were 1680 (25.2%) patients who described their satisfaction as excellent, 2574 (38.7%) very good, 1879 (28.2%) good, 382 (5.7%) fair, 96 (1.4%) poor, and 48 (0.7%) terrible. Overall, 6515 patients (97.8%) were satisfied with their operative outcomes.

On univariate analysis, male sex (mean [SD] satisfaction, 2.14 [0.97] [male] vs 2.24 [0.98] [female]; *P* = .001) and right-sided surgery (mean [SD] satisfaction, 2.19 [0.96] [right side] vs 2.24 [1.01] [left side]; *P* = .04) were significantly associated with greater satisfaction after surgery. On covariance analysis adjusting for sex and side of surgery, there was significantly greater patient satisfaction in patients with preoperative scores of 40 to less than 50 compared to those with scores of less than 20, 20 to less than 30, and 30 to less than 40. Patients with preoperative scores in the 50 or higher group appeared to be less satisfied compared with those in the 40 to less than 50 group, although the difference was not statistically significant. There were no other statistically significant differences between groups (Table 1).

**Table 1.** SF-36 LS Means by PCS Patient Satisfaction Group With Pairwise Comparisons Among PCS Groups

PCS Reference Group	SF-36 LS Mean (95% CI)	Comparison Group	LS Mean Difference	*P* Value
<20	2.23 (2.13-2.33)	20 to <30	0.04	.36
		30 to <40	0.06	.17
		40 to <50	0.16	.002
		≥50	0.12	.06
20 to <30	2.19 (2.11-2.26)	30 to <40	0.02	.47
		40 to <50	0.12	.001
		≥50	0.07	.14
30 to <40	2.17 (2.09-2.22)	40 to <50	0.09	.01
		≥50	0.05	.30
40 to <50	2.07 (1.98-2.16)	≥50	−0.04	.46
≥50	2.11 (2.00-2.23)		NA	NA

Abbreviations: LS, least squares; NA, not applicable; PCS, physical component summary; SF-36, 36-Item Short Form Health Survey.

Preoperative SF-36 PCS score was significantly negatively correlated with its ΔPCS (Spearman coefficient, –0.66; *y* = 42.05-0.81*x*; *P* < .001). A total of 4515 patients (67.8%) achieved the MCID of 10 from baseline. Patients achieving the MCID were significantly more satisfied than those who did not meet the MCID (2.12 vs 2.42; 95% CI, 0.25 ≤ Δμ ≤ −0.36; *P* < .001). From the dichotomized MCID outcome, we determined the optimal preoperative SF-36 PCS cutoff value to be 42.1 based on the Youden index. Area under the receiver operating characteristic curve was 0.80 (95% CI, 0.79-0.81). This cutoff corresponds to 50.0% sensitivity, 96.8% specificity, 84.8% positive predictive value, and 80.1% negative predictive value, with prevalence of no apparent improvement of 32.2%.

The SF-36 PCS cutoff of 42.1 was used to stratify the study population into estimated clinical responders (<42.1) and estimated nonresponders (≥42.1). Patient characteristics and outcomes of the corresponding subgroup analysis are shown in Table 2. Patients with higher preoperative SF-36 PCS tended to be male (327 of 1262 [25.9%] vs 1098 of 5397 [20.3%]) with lower BMI (27.0 vs 27.8) (both *P* < .001). Despite having significantly lower improvement (ΔPCS) scores (mean [SD], 2.67 [7.8] vs 19.0 [11.1]; *P* < .001), patients who were unlikely to meet the MCID had significantly higher 2-year SF-36 PCS scores (mean [SD], 51.1 [7.1] vs 47.5 [9.8]; *P* < .001) and greater satisfaction (mean [SD], 2.15 [0.9] vs 2.23 [1.0]; 95% CI, 0.02 ≤ Δμ ≤ 0.14; *P* = .009).

**Table 2.** Patient Characteristics and Outcomes of the Subgroup Analysis

Variable	Preoperative PCS <42.1 (n = 5397)	Preoperative PCS ≥42.1 (n = 1262)	*P* Value
Age, mean (SD), y	67.1 (7.9)	66.6 (7.0)	.07
Sex, No. (%)			
Male	1098 (20.3)	327 (25.9)	<.001
Female	4299 (79.7)	935 (74.1)	
Race/ethnicity, No. (%)			
Chinese	4603 (85.3)	1152 (91.3)	NA^a^
Malay	426 (7.9)	51 (4.0)	
Indian	307 (5.7)	50 (4.0)	
Other	61 (1.1)	9 (0.7)	
Side of surgery, No. (%)			
Left	2585 (47.9)	627 (49.7)	.03
Right	2812 (52.1)	635 (50.3)	
BMI, mean (SD)	27.8 (4.7)	27.0 (4.1)	<.001
Preoperative SF-36 PCS, mean (SD)^b^	28.5 (6.9)	48.4 (4.1)	<.001
ΔPCS, mean (SD)	19.0 (11.1)	2.67 (7.8)	<.001
2-y SF-36 PCS, mean (SD)	47.5 (9.8)	51.1 (7.1)	<.001
2-y satisfaction, mean (SD)^c^	2.23 (1.0)	2.15 (0.9)	.009

Abbreviations: BMI, body mass index (calculated as weight in kilograms divided by height in meters squared); NA, not applicable; PCS, physical component summary; SF-36, 36-Item Short Form Health Survey.

^a^ *P* value was not calculated for race/ethnicity owing to the large proportion of patients of Chinese race/ethnicity.

^b^ Range of possible scores, 0 to 100, with higher scores indicating better function.

^c^ Patient satisfaction was scored on a 6-level Likert scale (1 indicates excellent; 2, very good; 3, good; 4, fair; 5, poor; and 6, terrible).

The boxplot of patient satisfaction against postoperative SF-36 PCS revealed a decreasing mean postoperative SF-36 PCS with poorer patient satisfaction (Figure). There were considerable variance and outliers within each satisfaction group, with significant numbers of patients with either low satisfaction despite high scores or high satisfaction despite low scores.

**Figure.**
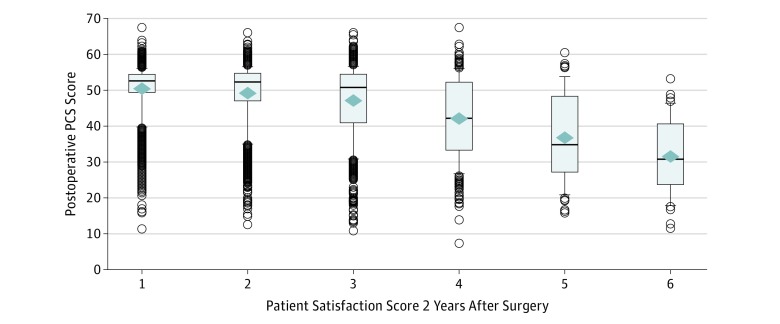
Boxplots of Patient Satisfaction Physical Component Summary (PCS) Score by 2-Year Postoperative Satisfaction Score on the 36-Item Short Form Survey Diamonds indicate the mean; lower and upper edges of the boxes represent the 25th and 75th percentiles (interquartile range [IQR]). The horizontal line indicates the median; the upper and lower extremes of the whiskers are defined as 1.5 × IQR above and 1.5 × IQR below the 75th and 25th percentiles. Circles identify the more extreme observations.

## Discussion

Total knee arthroplasty is typically indicated as a salvage option for end-stage arthrosis with reasonably good and predictable outcomes. Beyond an aging Baby Boomer generation and associated conditions, such as obesity, in the Western world, technological advances contributed to the ease and availability of TKA in most developed countries. This access led to TKA being performed on patients with less severe symptoms, ultimately increasing the volume of TKA performed.^1,3^

The inevitable increase in health care costs has led to a focus on value-based reimbursement as opposed to volume-based reimbursement. With that, measures of cost-effectiveness and patient-reported outcomes have become important considerations in funding health care procedures. However, health care costs differ among systems and countries. In a system with lower-cost access to health care, the cutoff for cost-effectiveness should correspondingly be higher. Patient expectations are also known to differ among cultural and racial/ethnic groups.^14,15,16,17^ Thus, a cutoff determined by a certain patient demographic characteristic might not be translatable owing to cost and population differences. Furthermore, owing to variations in costs, disability below the cutoff as defined based on cost-effectiveness alone does not necessarily imply that no medical or psychological benefits can be derived from the procedure at the individual patient level. In our study, despite significantly lower score improvements, our patients with preoperative SF-36 PCS of 42.1 or greater were more satisfied with their operative outcomes. Patient satisfaction was high, with little variability among the different bands of preoperative scores. Although all health care systems are realistically on a spectrum in health economics, hypothetically, in a world of unlimited resources, overreliance on score outcomes for decision making may deprive patients of access to quality health care.

Patient-reported outcome measures have become a cornerstone of outcome assessment after joint surgery.^18^ However, the interpretation and utility of these measures in clinical practice remains debatable.^18,19,20,21^ In a study on patients after total hip arthroplasty, Rogers et al^20^ reported that the preoperative Western Ontario and McMaster Universities (WOMAC) score does not predict the postoperative WOMAC score or patient satisfaction after surgery. Likewise, our study found that preoperative scores do not correlate well with patient satisfaction after surgery. The MCID is a more clinically oriented concept that attempts to go beyond statistically significant changes at the group level that may not be significant at the individual level to find the smallest difference between the questionnaire scores that the patient perceives to be beneficial.^13,22^ Although Ferket et al^4^ identified a preoperative SF-36 PCS cutoff based on cost-effectiveness only on score improvement, we further identified the cutoff that was associated with the individual patient experiencing MCID. However, this cutoff did not translate into better patient satisfaction. The best satisfaction was seen in the group with preoperative scores of 40 to less than 50. Our findings support the assertion that feeling better does not always equate to feeling good and that improvements in outcome scores, however large, do not necessarily indicate acceptability of the current state.^19^

In our study, despite only meeting the MCID for SF-36 PCS in 67.8% of patients, overall patient satisfaction was high at 97.8%. Despite a negative correlation between preoperative SF-36 PCS and its Δ score, counterintuitively, patients with good preoperative PCS still had better satisfaction rates compared with those in the other bands. Moreover, a preoperative SF-36 PCS of 42.1 or greater was significantly associated with better patient satisfaction after TKA despite our finding that, in patients with such high preoperative scores, MCID was unlikely to be achieved. Given these considerations, the discordance in findings may be attributed to 32.2% of patients being satisfied with surgery despite not achieving an MCID. This is further supported by the analysis of postoperative scores that revealed a significant variance and outliers within each satisfaction group. Because patient-reported outcome scores reduce the patient symptoms and circumstances to a digit, predictive models based on disability alone may fail to consider the individual patient symptoms and circumstances.

Patient satisfaction with TKA is a multifactorial concept. Statistically significant outcome improvement at the group level may not necessarily translate to patient satisfaction at the individual level. The SF-36 survey, moreover, does not specifically assess patient satisfaction. In determining patient satisfaction, patients are more likely to focus on their present state of health than to consider the extent of improvement that they have experienced.^23,24^ Higher absolute postoperative scores and meeting patient expectations are more consistent determinants of patient satisfaction.^25,26,27^ Likewise, we found that postoperative SF-36 PCS scores better correlated with patient satisfaction. Knee-specific scores, such as WOMAC, Oxford, and Knee Society Scores, may also be helpful in quantifying functional ability. However, these scores will not replace a methodical history taking, comprehensive physical examination, and thorough discussion with the patient with careful understanding of his or her expectations and goals with surgery. In determining the cost-effectiveness of TKA in addition to knee function, the general health and emotional state of the patient should be considered holistically to estimate his or her final functional status after surgery.^25^ Future studies looking into this interplay of factors may allow us to identify novel factors associated with high absolute postoperative scores as a surrogate for patient satisfaction.

### Limitations

Our study is limited in the following areas. First, to quantify satisfaction, our center used a 6-level Likert scale, with lower scores indicating better outcomes, adapted from the North American Spine Society questionnaire. A simple question may lack the sensitivity in detecting subtle differences accurately compared with a comprehensive questionnaire. Patients also have different opinions on what constitutes excellent and very good responses. We were also unable to delineate the specific reasons behind patient satisfaction or dissatisfaction. However, comprehensive questionnaires tend to be vulnerable to bias. Robertsson et al^28^ reported the usable return rate of a more comprehensive questionnaire to be 18% to 45% lower than that of a simple satisfaction questionnaire. Patients not responding to the comprehensive questionnaires were more often unsatisfied with their operated knee than patients responding.

Second, our study looked into the use of only the SF-36 PCS in measuring patient satisfaction. We are unable to comment on whether a combination of patient-reported outcomes (such as in conjunction with the Knee Society Score and the Oxford Knee Score) will have a stronger correlation with patient satisfaction. Further studies will be helpful in elucidating this correlation.

Third, our study focused on the early 2-year outcomes and satisfaction. We did not have the data from longer-term (5-year and 10-year) follow-up. Likewise, we were unable to state whether our findings could be extrapolated accordingly. Anecdotally, the decline in functional ability after the 2-year mark in our local context is often a result of developing lumbar degenerative conditions. Because it can be challenging to objectively quantify this disability, the 2-year mark was specifically chosen because it approximates the time when early rehabilitation potential plateaus with concurrent reduction in the likelihood of an unrelated musculoskeletal event affecting the SF-36 scores with prolonged follow-up.

## Conclusions

The findings suggest that a general health score, such as the SF-36, does not correlate well with patient satisfaction after TKA. Good postoperative SF-36 scores were generally indicative of better patient satisfaction. Higher preoperative PCS scores were associated with lower potential for improvement after TKA; however, they were associated with higher overall satisfaction, despite smaller interval improvements. The utility of SF-36 PCS as a preoperative adjunct in an assessment of surgery remains unclear. Functional assessment, preoperative counseling, and modification of expectations may be vital before TKA surgery.
